# Nerve Growth Factor Regulation by TNF-*α* and IL-1*β* in Synovial Macrophages and Fibroblasts in Osteoarthritic Mice

**DOI:** 10.1155/2016/5706359

**Published:** 2016-08-18

**Authors:** Shotaro Takano, Kentaro Uchida, Masayuki Miyagi, Gen Inoue, Hisako Fujimaki, Jun Aikawa, Dai Iwase, Atsushi Minatani, Kazuya Iwabuchi, Masashi Takaso

**Affiliations:** ^1^Department of Orthopedic Surgery, Kitasato University School of Medicine, 1-15-1 Minami-ku Kitasato, Sagamihara City, Kanagawa 252-0374, Japan; ^2^Department of Immunology, Kitasato University School of Medicine, 1-15-1 Minami-ku Kitasato, Sagamihara City, Kanagawa 252-0374, Japan

## Abstract

To investigate the role of macrophages as a regulator and producer of nerve growth factor (NGF) in the synovial tissue (ST) of osteoarthritis (OA) joints, the gene expression profiles of several inflammatory cytokines in the ST, including synovial macrophages and fibroblasts, of OA mice (STR/Ort) were characterized. Specifically, real-time polymerase chain reaction analysis was used to evaluate the expression of tumor necrosis factor- (TNF-) *α*, interleukin- (IL-) 1*β*, IL-6, and NGF in CD11b+ and CD11b– cells isolated from the ST of a murine OA model. The effects of TNF-*α*, IL-1*β*, and IL-6 on the expression of NGF in cultured synovial cells were also examined. The expression of TNF-*α*, IL-1*β*, IL-6, and NGF in the ST of STR/Ort was higher than that in C57/BL6J mice. Compared to the CD11b– cell fraction, higher expression levels of TNF-*α*, IL-1*β*, and IL-6 were detected in the CD11b+ cell fraction, whereas no differences in the expression of NGF were detected between the two cell fractions. Notably, TNF-*α* upregulated NGF expression in synovial fibroblasts and macrophages and IL-1*β* upregulated NGF expression in synovial fibroblasts. IL-1*β* and TNF-*α* may regulate NGF signaling in OA joints and be suitable therapeutic targets for treating OA pain.

## 1. Introduction

Osteoarthritis (OA) is characterized by cartilage breakdown, synovial fibrosis, and osteophyte formation. The main clinical symptom of OA is chronic joint pain, which is typically treated using analgesic drugs, such as nonsteroidal anti-inflammatory drugs and corticosteroids [1, 2]. However, due to potential adverse effects [3, 4] and variable efficacy of these drugs for providing symptomatic pain relief, more effective analgesics are needed to improve OA treatment and patient care. In addition, because only a few effective disease-modifying drugs are available for OA [5, 6], a better understanding of the mechanisms that drive OA pain is required to guide drug development.

Nerve growth factor (NGF) is a widely known pain mediator [7] and plays a critical role in the modulation of OA pain [8–11]. The neutralization of NGF with tanezumab, an anti-NGF monoclonal antibody, has robust analgesic effects on OA pain [8, 10, 11]. Due to these effects, the regulation of NGF has been investigated in several* in vitro* and* in vivo* studies [12, 13]. Evidence from these studies suggests that the activity of NGF is mediated by inflammatory cytokines. For example, in an experimental arthritic mouse model, IL-1*β*, but not TNF-*α*, increased NGF levels in knee joints [13]. However, the mechanism regulating NGF expression in synovial tissue (ST) remains unclear.

ST contains macrophage- and fibroblast-like cells in the lining layer [14–16]. The treatment of cultured synovial OA fibroblasts with IL-1*β* and TNF-*α* induces the production of NGF [12]. More recent studies have shown that macrophages produce a number of inflammatory cytokines, including IL-1*β*, IL-6, and TNF-*α*, which contribute to OA progression and associated joint pain [17–21]. Despite these findings, the regulation of NGF expression in synovial macrophages is not fully understood.

STR/Ort mice are a well-characterized OA model that spontaneously develop OA with a progression resembling that of humans [22–24]. Synovial hyperplasia is also observed in STR/Ort mice [25], and we previously showed that the CD11b+ macrophage population in ST STR/Ort mice is higher than that found in C57BL/6J mice [17, 18]. In addition, we reported that compared to synovial fibroblasts (CD11b−), synovial macrophages produce high levels of IL-1*β* and TNF-*α* in STR/Ort mice [18].

Here, we characterized the expression profiles of several inflammatory cytokines and NGF in the ST of STR/Ort mice. In addition, the regulation of NGF expression by inflammatory cytokines in synovial macrophages and fibroblasts was also examined.

## 2. Materials and Methods

### 2.1. Animals

Nine-month-old male STR/Ort (average body weight, 40.6 ± 4.1 g) and C57BL/6J (control; average body weight, 32.8 ± 1.2 g) mice (Charles River Laboratories, Inc., Yokohama, Japan) were used in this study. Specific pathogen-free colonies of each mouse line were maintained at Nippon Charles River Laboratories (Kanagawa, Japan) in a semibarrier system with a controlled environment (temperature: 23 ± 2°C; humidity: 55% ± 10%; lighting: 12 h light/dark cycle). All experimental protocols were approved by the Kitasato University School of Medicine Animal Care Committee.

### 2.2. Expression of Inflammatory Cytokines and NGF in ST

C57BL/6J and STR/Ort mice were sacrificed by the intramuscular injection of a mixture of medetomidine, midazolam, and butorphanol tartrate. After removing skin with a scalpel, ST was harvested, and total RNA was then extracted from the harvested tissue using TRIzol (Invitrogen, Carlsbad, CA, USA), according to the manufacturer's instructions. The extracted total RNA was used as a template for first-strand cDNA synthesis using SuperScript III RT (Invitrogen) in PCR reaction mixtures consisting of 2 *μ*L cDNA, specific primer set (0.2 *μ*M final concentration), and 12.5 *μ*L SYBR Premix Ex Taq (Takara, Kyoto, Japan) in a final volume of 25 *μ*L. The primers for IL6 and NGF were designed using Primer Blast software and were synthesized by Hokkaido System Science Co., Ltd. (Sapporo, Japan). The other primers used in this study were designed based on previously published primer sequences [17]. The sequences of the PCR primer pairs used in this study are listed in Table 1. The specificity of the amplified products was confirmed by melt curve analysis. Quantitative PCR was performed using a CFX-96 real-time PCR detection system (Bio-Rad, Hercules, CA, USA). The PCR cycles consisted of an initial denaturation step at 95°C for 1 min, followed by 40 cycles of 95°C for 5 s and 60°C for 30 s. mRNA expression was normalized to the levels of glyceraldehyde-3-phosphate dehydrogenase (GAPDH) mRNA. The gene expression levels in the ST of STR/Ort mice were compared with those in the ST of C57BL/6J mice (STR/Ort/C57BL6J). In addition, the gene expression levels in CD11b+ cells were compared with those in CD11b− cells (CD11b+/CD11b−).

### 2.3. Isolation of CD11b-Positive and CD11b-Negative Cells from ST

ST samples were collected from both knees of five STR/Ort mice, and mononuclear cells were then isolated from the collected tissue by digestion with type I collagenase for 2 h at 37°C. The obtained mononuclear cells were suspended in 500 *μ*L phosphate-buffered saline (PBS) containing biotinylated anti-CD11b antibody. Following a 30 min incubation at 4°C, the cells were washed with PBS, mixed with streptavidin-labelled magnetic particles (BD IMag Streptavidin Particles Plus-DM; BD Biosciences, Tokyo, Japan), and incubated for 30 min on ice in an IMag separation system (BD Biosciences). Warmed (37°C) *α*-Minimum Essential Medium (MEM) was added to the cell suspension to collect unbound (CD11b-negative) cells, and an additional 3 mL *α*-MEM was added to collect CD11b-positive cells after removing the tub from the magnetic support. The CD11b-positive and CD11b-negative cells were collected by centrifugation at 300 ×g for 10 min, and the obtained cells were cultured in *α*-MEM in six-well plates. TNF-*α*, IL-1*β*, IL-6, and NGF expression in the cells was analyzed by reverse transcription-polymerase chain reaction (RT–PCR). The experiment was performed four times independently.

### 2.4. Effect of Cytokines on ST-Derived Cells

ST-derived mononuclear cells, including synovial macrophages and fibroblasts, which were isolated as described above, were cultured in *α*-MEM in six-well plates. After a 1-week incubation at 37°C in a 5% CO_2_ incubator, synovial fibroblasts were incubated with either mouse recombinant TNF-*α* (25 ng/mL), IL-1*β* (50 ng/mL), or IL-6 (100 ng/mL) (Biolegend, San Diego, CA, USA) for 24 h. Cells that were not treated with any cytokines were used as controls. To examine the effect of TNF-*α* on synovial macrophages and fibroblasts, CD11b-positive and CD11b-negative cells were collected from ST, as described above, and were then cultured in *α*-MEM in six-well plates. After a 1-week incubation at 37°C in a 5% CO_2_ incubator, CD11b-positive and negative cells were incubated with mouse recombinant TNF-*α* (25 ng/mL) or IL-1*β* (50 ng/mL) for 24 h. Cells that were not treated with TNF-*α* were used as controls. The treated and control cells were harvested for total RNA isolation, as described above, and NGF expression was analyzed by RT–PCR. The experiment was performed four times independently.

### 2.5. Immunohistochemical Analysis of Dorsal Root Ganglia (DRG) Specimens

Ten L3 and L4 DRG samples were harvested from the 9-month-old C57BL/6J and STR/Ort mice and were then immersed in a buffered paraformaldehyde fixative at 4°C overnight. The samples were further incubated in PBS containing 20% sucrose for 24 h at 4°C. After freezing in liquid nitrogen, each specimen was sectioned at 10 *μ*m thickness on a cryostat (Leica Microsystems, CM3050S, Wetzlar, Germany) and was then treated for 90 min at room temperature with a blocking solution of PBS containing 0.05% Tween-20 and 1% skim milk.

The sectioned DRG specimens were stained with rabbit antibodies against calcitonin gene-related peptide (CGRP; 1 : 1000; Immunostar, Hudson, WI, USA) and transient receptor potential vanilloid 1 (TRPV1; 1 : 500; Calbiochem, San Diego, CA, USA) by incubation for 18 h at 4°C. The DRG sections were then incubated with Alexa 488-conjugated goat anti-rabbit IgG (for CGRP immunoreactivity, 1 : 1000; Molecular Probes) and Alexa 488-conjugated goat anti-rabbit IgG (for TRPV1 immunoreactivity, 1 : 1000; Molecular Probes). The immunostained sections were visualized using a fluorescence microscope (Axiovert 200, Zeiss, Jena, Germany) in a treatment-blinded manner. The numbers of CGRP- and TRPV-positive cells were counted, and the proportion of these cells to the total number of nucleated cells in DRG was calculated for each DRG sample.

### 2.6. Statistical Analysis

Differences between C57BL/6J and STR/Ort mice were examined using the* t*-test. All statistical analyses were performed with SPSS software (Version 11.0; SPSS, Inc., Chicago, IL, USA). A *P* value of < 0.05 was considered statistically significant.

## 3. Results

### 3.1. Expression of TNF-*α*, IL-1*β*, IL-6, and NGF in ST

**Figure 1:**
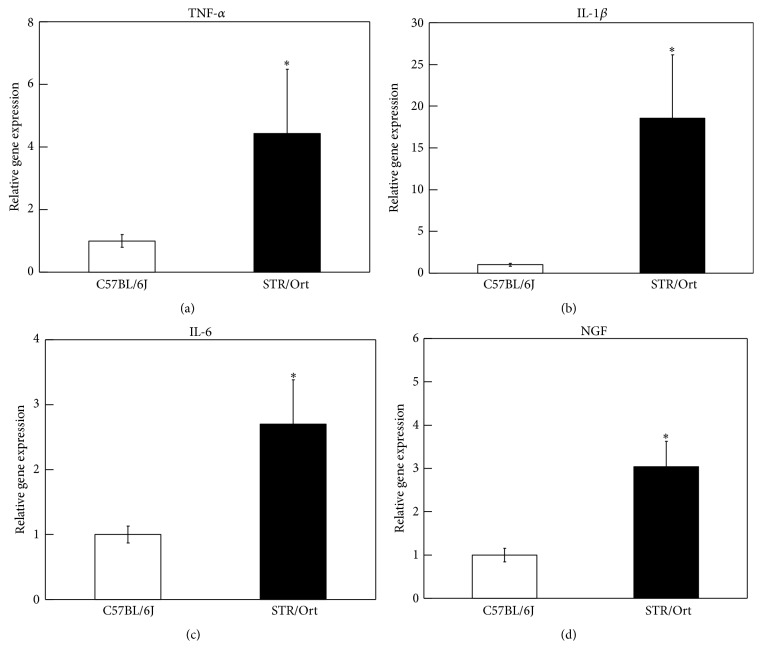
Real-time PCR analysis for the gene expression of TNF-*α*, IL-1*β*, IL-6, and NGF in synovial tissues (a–d) of C57BL/6J and STR/Ort mice. *∗* indicates a statistically significant difference between C57BL/6J and STR/Ort mice. All data are presented as the mean ± SE (*n* = 10).

Real-time PCR analysis of the genes encoding TNF-*α*, IL-1*β*, IL-6, and NGF showed that the expression levels of these genes were significantly elevated in the ST of STR/Ort mice compared to those in control C57BL/6J mice (Figures 1(a)–1(d)).

**Figure 2:**
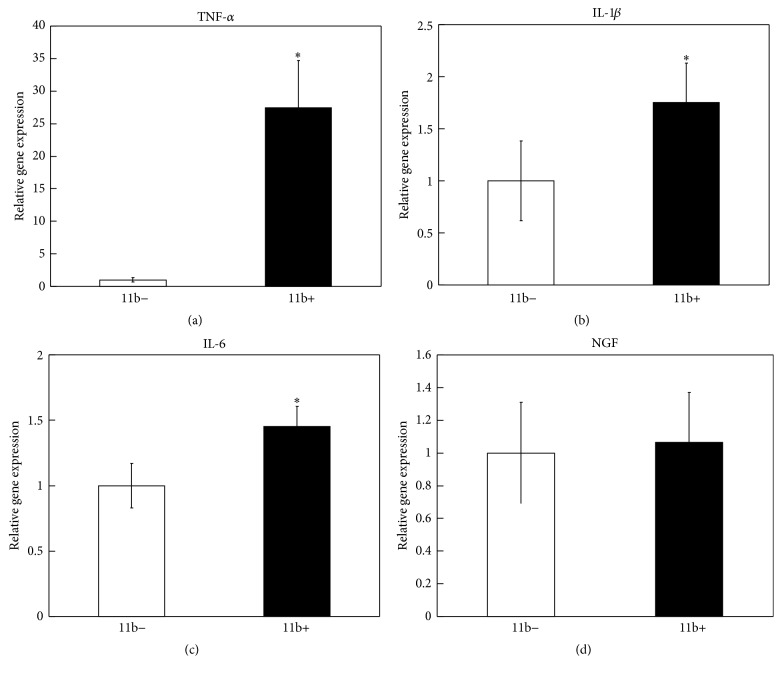
Real-time PCR analysis for the gene expression of TNF-*α*, IL-1*β*, IL-6, and NGF in CD11b-negative and CD11b-positive cell fractions obtained from synovial tissue of STR/Ort mice. All data are presented as the mean ± SE of four experiments.

Activated macrophages reportedly produce higher levels of inflammatory cytokines, including TNF-*α*, IL-1*β*, and IL-6, than tissue-resident macrophages [18, 26, 27]. To determine whether macrophages in ST also produce inflammatory cytokines, expression of the genes encoding TNF-*α*, IL-1*β*, and IL-6 in CD11b-positive cells isolated from the ST of STR/Ort mice was examined by real-time PCR. TNF-*α*, IL-1*β*, and IL-6 gene expression in CD11b-positive cell fractions was higher than that in CD11b-negative cell fractions (Figures 2(a)–2(c)). In contrast, no differences in the gene expression of NGF were detected between the two examined cell fractions (Figure 2(d)).

### 3.2. Effect of TNF-*α*, IL-1*β*, and IL-6 on NGF Expression in Synovial Cells

**Figure 3:**
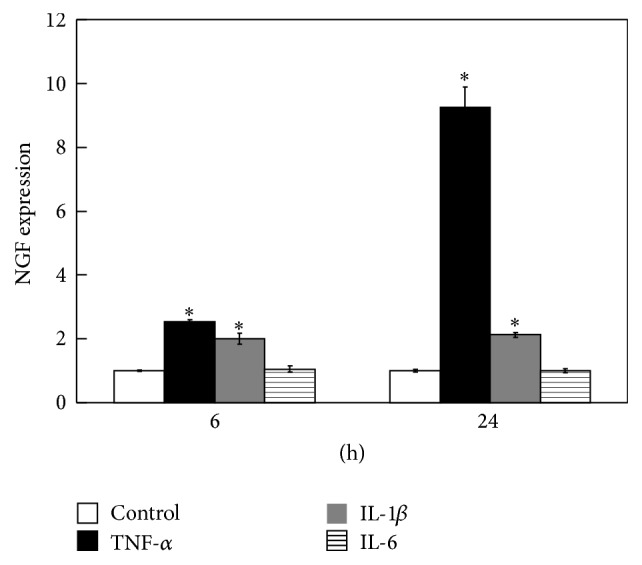
Effect of inflammatory cytokines on NGF gene expression in cultured synovial cells. Effect of TNF-*α*, IL-1*β*, and IL-6 on NGF gene expression in cultured synovial cells obtained from STR/Ort mice. All real-time PCR data are presented as the mean ± SE of four experiments. Cells stimulated with cytokines were compared with untreated cells as a control. ^*∗*^ *p* < 0.05 versus control group.

**Figure 4:**
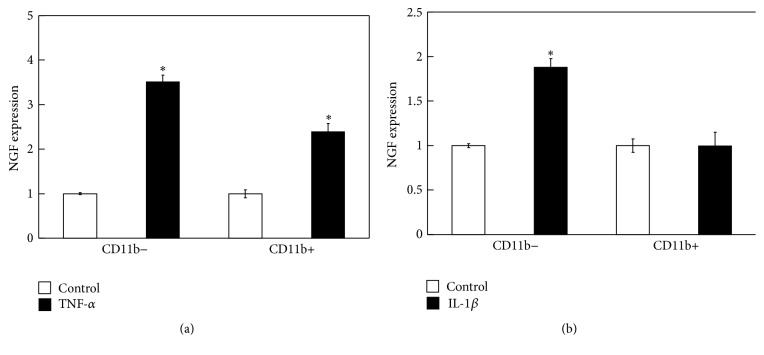
Effect of inflammatory cytokines on NGF gene expression in cultured synovial fibroblasts and macrophages. Effect of TNF-*α* (a) and IL-1*β* (b) on NGF gene expression in CD11b-positive and CD11b-negative cell fractions obtained from STR/Ort mice. ^*∗*^ *p* < 0.05 versus control group.

Real-time PCR analysis revealed that the gene expression of NGF increased significantly in isolated synovial cells in the presence of exogenously added TNF-*α* and IL-1*β* compared to untreated control cells (Figure 3). In contrast, the gene expression of NGF was not affected in IL-6-treated synovial cells (Figure 3). NGF expression was also significantly increased in the presence of exogenously added TNF-*α* in isolated populations of synovial fibroblasts and macrophages compared to untreated control cells (Figure 4(a)). NGF expression was also significantly increased in the presence of exogenously added IL-1*β* in isolated populations of synovial fibroblasts, but not in synovial macrophages (Figure 4(b)).

### 3.3. Immunohistochemical Analysis of DRG

**Figure 5:**
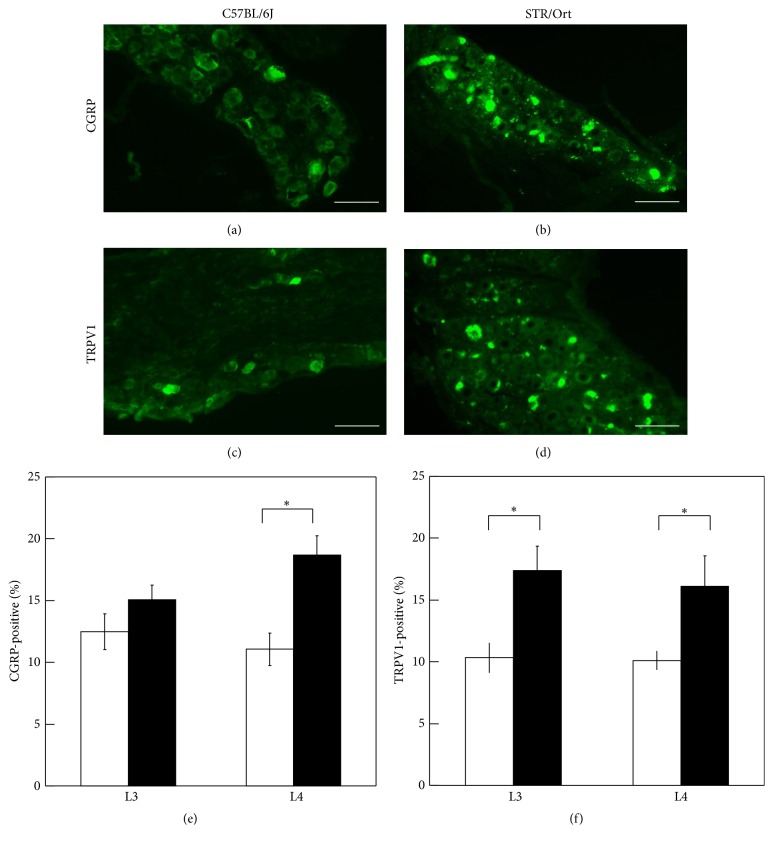
Expression of CGRP and TRPV1 in dorsal root ganglia. Expression of CGRP in L4 dorsal root ganglia of C57BL/6J (a) and STR/Ort mice (b). Expression of TRPV1 in L4 dorsal root ganglia of C57BL/6J (c) and STR/Ort mice (d). Scale bar = 100 *μ*m. Percentage of CGRP-positive (e) and TRPV1-positive (f) cells in dorsal root ganglia of C57BL/6J and STR/Ort mice. Ten C57BL/6J and STR/Ort mice were used for this experiment. Data are presented as the mean ± SE (*n* = 10). ^*∗*^ *p* < 0.05 versus C57BL/6J.

To investigate pain-related sensory innervation by NGF, the proportion of TRPV1- and CGRP-positive cells in the DRG of STR/Ort mice was investigated by immunohistochemistry. The number of TRPV1- (Figures 5(a), 5(b), and 5(e)) and CGRP-positive cells (Figures 5(b), 5(d), and 5(f)) in the DRG of STR/Ort mice was significantly higher compared to that found in the DRG of C57BL/6J mice.

## 4. Discussion

In the present study investigating the mechanisms underlying the regulation of NGF and development of OA pain, higher expression of the genes encoding TNF-*α*, IL-1*β*, IL-6, and NGF was observed in the ST of an OA STR/Ort mouse model compared to that in C57BL/6J mice. In addition, NGF expression was specifically detected in the CD11b-positive and CD11b-negative cell fractions isolated from ST, whereas higher TNF-*α*, IL-1*β*, and IL-6 gene expression was observed in the CD11b-positive cell fractions. Notably, the treatment of cultured synovial cells with TNF-*α* and IL-1*β* stimulated NGF expression and TNF-*α* also stimulated NGF expression in CD11b-positive and CD11b-negative cell fractions. The number of TRPV1- and CGRP-positive cells in the DRG of STR/Ort mice was significantly higher compared to that found in the DRG of C57BL/6J mice. Taken together, these findings suggest that TNF-*α* regulates NGF expression in both synovial fibroblasts and macrophages and that IL-1*β* regulates NGF expression in synovial fibroblasts and that these factors may contribute to the development of pain in OA patients by innervation of the peripheral nervous system.

NGF is upregulated in human synovial fibroblasts, suggesting that this factor plays an important role in OA pathology [12, 13, 28]. NGF elevated inflammatory knee joint and TNF-*α* and IL-1*β* stimulate synovial fibroblasts to produce NGF. Manni et al. [12] reported that NGF expression is upregulated in the knee joint of a carrageenan-treated inflammatory arthritis model and is further stimulated by the intraarticular injection of TNF-*α* and IL-1*β* [13]. Further, TNF-*α* and IL-1*β* treatment of cultured synovial fibroblasts derived from a human OA patient also increased NGF production [12]. However, because these studies consisted of analyses of whole knee joints or cultured synovial fibroblasts exogenously stimulated with TNF-*α* and IL-1*β*, the cell populations responsible for regulating NGF and producing TNF-*α* and IL-1*β* in ST were not conclusively determined. Here, elevated expression of the inflammatory cytokines TNF-*α*, IL-1*β*, and IL-6 was observed in the ST of STR/Ort mice, and TNF-*α* and IL-1*β* were increased in synovial macrophages isolated from this OA model. In addition, NGF expression was stimulated by TNF-*α* and IL-1*β* in the synovial fibroblast fraction. Taken together, these findings suggest that TNF-*α* and IL-1*β* stimulate NGF expression in synovial fibroblasts.

A recent immunohistochemical analysis revealed that NGF not only is localized to fibroblasts, but is also produced by a specific population of macrophages in human ST [29]. However, the factor regulating NGF in macrophages was not determined. In the present study, isolated synovial macrophages and fibroblasts from STR/Ort mice expressed NGF at similar levels to each other. NGF expression in the macrophage fraction was also stimulated by TNF-*α*. Taken together, these findings indicate that TNF-*α* stimulates NGF expression in both synovial fibroblasts and macrophages.

NGF plays a key role in the generation of acute and chronic pain and peripheral sensitization [30–33]. Recent evidence suggests that the TRPV1 ion channel and CGRP are involved in the development of NGF-induced persistent mechanical and thermal hypersensitivity in rats [30, 31]. NGF acutely modulates TRPV1 activity [32, 33] and also stimulates CGRP expression in DRG neurons* in vitro* and* in vivo* [34]. Consistent with this speculation, inhibition of NGF by treatment with tanezumab was recently shown to have high analgesic efficacy in OA knees compared with placebo [8]. In the present study, CGRP- and TRPV1-positive cells, which are localized in DRG neurons in monoiodoacetate- (MIA-) treated OA animals [35, 36], were increased in the DRG of STR/Ort compared to C57BL/6J mice, suggesting that the elevation of synovial NGF in STR/Ort mice contributes to peripheral sensitization. However, several studies have shown that human OA chondrocytes also produce NGF by inflammatory cytokines [37, 38]. Further investigations are needed to clarify the contribution of chondrocytes derived NGF on peripheral sensitization.

Several limitations of the present study warrant mention. First, the mechanism underlying the development of OA pain that was proposed based on the present results was based on cross-sectional analysis and therefore no data on the effects of the observed changes on the progression of OA are available. Second, although NGF was found to be elevated in the ST of OA mice, it remains to be determined if the elevation of synovial NGF levels contributes to peripheral sensitization. Finally, present study did not confirm that the protein levels of these cytokines correlated with the expression levels.

In conclusion, the present results support the hypothesis that TNF-*α* regulates NGF expression in synovial fibroblasts and macrophages and IL-1*β* regulates NGF expression in synovial fibroblasts in OA mice. TNF-*α* and IL-1*β* may therefore regulate NGF signaling in OA joints and be suitable therapeutic targets for treating OA pain.

## Figures and Tables

**Table 1:** Sequences of the primers used in this study.

Primer	Sequence (5′-3′)	Product size (bp)
TNF-*α*-F	CTGAACTTCGGGGTGATCGG	122
TNF-*α*-R	GGCTTGTCACTCGAATTTTGAGA	

IL-1*β*-F	GCAACTGTTCCTGAACTCAACT	89
IL-1*β*-R	ATCTTTTGGGGTCCGTCAACT	

IL-6-F	CTGCAAGAGACTTCCATCCAG	131
IL-6-R	AGTGGTATAGACAGGTCTGTTGG	

NGF-F	ATGGTGGAGTTTTGGCCTGT	192
NGF-R	GTACGCCGATCAAAAACGCA	

GAPDH-F	AACTTTGGCATTGTGGAAGG	223
GAPDH-R	ACACATTGGGGGTAGGAACA	
